# Laboratory simulation of the swampy forest system for the passive treatment of acid mine drainage in coal mine reclamation areas

**DOI:** 10.1038/s41598-023-32990-x

**Published:** 2023-04-13

**Authors:** Ihsan Noor, Yudi Firmanul Arifin, Bambang Joko Priatmadi, Akhmad Rizalli Saidy

**Affiliations:** 1 Mining Engineering Study Program, Faculty of Engineering, Lambung Mangkurat University, Jalan Jenderal Ahmad Yani KM-34, Banjarbaru, South Kalimantan Indonesia 70714; 2 Mine Closure Research Center, Lambung Mangkurat University, Banjarbaru, South Kalimantan, Indonesia 70714; 3 Sustainable Development Goals (SDGs) Center, Lambung Mangkurat University, Banjarbaru, South Kalimantan Indonesia 70714; 4 Forestry Science Study Program, Faculty of Forestry, Lambung Mangkurat University, Banjarbaru, South Kalimantan Indonesia 70714; 5 Center of Excellence for Innovation, Technology, Commercialization, Management: Forest and Wetland of Lambung Mangkurat University, Banjarbaru, South Kalimantan Indonesia 70714; 6 Soil Science Study Program, Faculty of Agriculture, Lambung Mangkurat University, Banjarbaru, South Kalimantan Indonesia 70714

**Keywords:** Ecology, Ecology, Environmental sciences, Engineering

## Abstract

Coal mining that applies the open pit method has the potential to generate acid mine drainage. Acid mine drainage (AMD) treatments must include processes to mitigate significant challenges; these treatments include active treatment with high costs and process uncertainty and passive treatment with its limitations. The new concept of the swampy forest system involves the development of passive treatment for AMD, which lowers costs, increases capacity, and provides a natural process to mitigate the AMD that has been generated. A laboratory simulation experiment was carried out to obtain the basic data required for the swampy forest system treatment. The basic reference data determined in this study, including the total volume of water, the water debt flows into the swampy forest scale laboratory system and the retention time, were obtained to bring the parameter values that failed to meet the quality standards into compliance, according to the applicable regulations. The AMD swampy forest treatment design in the pilot project at the treatment field can apply a scaled-up version of the basic data from the simulation laboratory experiment results.

## Introduction

Coal mining in Indonesia mostly applies the open pit mining method. Coal mining with the open pit mining method has the potential to cause several environmental impacts in the form of water, soil, and air if it is not managed correctly based on applicable regulations. During mining operations, land clearing and overburden (OB) removal cause the oxidation of pyrite minerals which can lead to the formation of AMD^1^. The AMD that has already occurred must be managed properly, especially drainages that are accommodated in sumps (during operation) or in voids (during mine closure stage) before the wastewater is released into public water bodies^8,27^. AMD may be formed by potential acid forming (PAF) material factors and can interfere with the growth of the revegetation during the reclamation process^11,21^.

There are two techniques in AMD management; prevention techniques by controlling the source of generated AMD and remediation techniques as a mitigation process for previously produced AMD^24^. In terms of AMD remediation, active treatments are generally considered more costly than passive treatments, especially when mining operations have ceased or mines have been abandoned^3^.

Gautama et al.^7^, have conducted a study on the use of lime for the neutralization process at pit lakes namely M23E in a coal mine in South Kalimantan. The amount of acidic wastewater in this pit lake was about 8.2 million m^3^. This study reviewed the implementation of active treatment in the form of in-pit treatment methods that have been carried out in the pit lake. Field-scale experimental processing uses lime to neutralize around 460,000 m^3^ of liquid waste which has a pH of 2.8 to be pH > 7 in three months before being released into public waters. The costs incurred are around USD 0.04 per m^3^ of acid water to meet quality standards. The coal mine has another about 85 million acid water that must be managed so it will be very expensive if it is still using active treatment.

Passive treatment systems can be a component of an AMD treatment strategy. They can function as either stand-alone treatment strategies or as pretreatment to reduce the cost of active treatment. Passive treatment system performance varies significantly among constructed systems, due to differences in site conditions^26^. Passive treatment methods generally achieve precipitation of metal sulfide by creating reducing conditions and utilizing organic substances as alkaline agents, including aerobic constructed wetlands and compost reactors^14^. Constructed wetlands are a promising passive treatment option because they are relatively self-sustaining once established and are deemed to be cost effective^17,24^. Passive treatment systems generally require longer retention times and greater space but markedly decrease long-term costs.

AMD must be managed better refers to the permit given to the coal mining that the wastewater must be treated to meet the environmental quality standards that have been set before release to the public rivers. Management of AMD must be carried out both prevention and treatment when the AMD has been formed. Companies that dispose of their wastewater without comply with the threshold quality standards will receive the administrative sanctions up to the suspension of the permits and criminal sanctions referring to existing regulations but most of active treatment more expensive than the passive treatment. As the passive treatment need more time and space.

We have developed the swampy forest system as a new natural method of passive AMD treatment with lower costs and greater environmental sustainability. The swampy forest system consists of selecting organic matter and combining it with the planting of selected grass and tree species in the form of a forest constructed wetland^17^. As a preliminary step in the swampy forest system, a laboratory experiments were carried out to combine the three materials that were selected before application to the pilot project. The aim of the present study was to rapidly decide the basic reference data for application to the next stage of the pilot project, which will be accomplished via a field study for a larger area with higher capacity^17^. The basic reference data that will be determined in this study, including the total volume of water, the water debt flows into the swampy forest scale laboratory system and the retention time, are obtained to change the parameter values that do not meet the quality standards to those that do meet them, according to the applicable regulations^17^.

## Materials and methods

### Experimental design

The role of each individual material used as the main ingredient for the swampy forest system was determined by batch reactor experiments carried out in a previous study. In the experiment of organic matter selection, the waste of oil palm (empty fruit bunches) was determined to increase the pH value from < 4, which did not meet the quality standard, to pH 6 – 9, which does meet the quality standard^18^. Grass species selection experiments determined the types of local grasses, namely, *Eleocharis dulcis* (purun grass) and *Cyperus rotondus* (batibati grass), while the nonlocal grass species are *Typha angustifolia* (typha grass) and *Vetiveria zizaniodes* (vetiver grass)^19^. Tree species selection experiments determined the type of local tree species, namely, *Melaleuca leucadendra* (galam tree) and *Nauclea subdita* (bangkal tree), while nonlocal tree species are *Nauclea orientalis* (longkida tree) and *Melaleuca cajuputi* (kayuputih tree)^20^. The experiment design mentioned in Table 1.

**Table 1:**
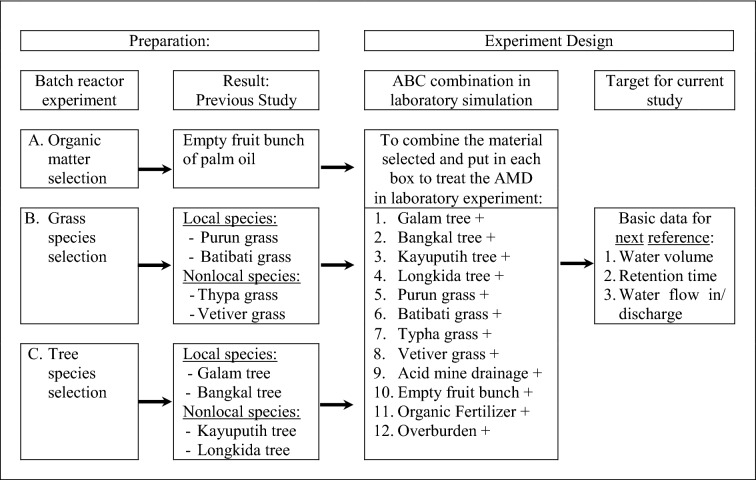
Experimental design for laboratory simulation.

**Figure 1:**
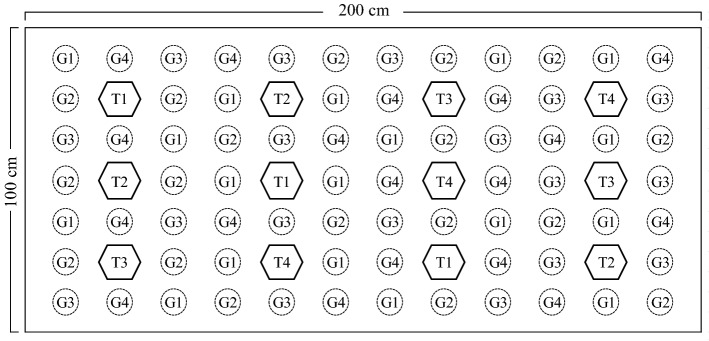
Grass and tree planting design for each box experiment (G1 = purun grass, G2 = typha grass, G3 = batibati grass, G4 = vertiver grass, T1 = galam tree, T2 = longkida tree, T3 = bangkal tree, T4 = kayuputih tree).

The results of the material selections noted above were used for the next combination experiment, namely, a laboratory simulation experiment intended to develop a passive treatment with a swampy forest system using the concept of a forest construction wetland^17^. Laboratory simulation experiments were performed by preparing an experimental reactor with a length 200 cm x width of 100 cm and height of 60 cm. This experiment was carried out in the reclamation land ex pit of a coal mining company (JBG) in South Kalimantan, Indonesia by preparing three experimental boxes connected in series. The experimental design for planting grass and tree species in each box is illustrated in Fig. 1.

### Experimental procedure

**Figure 2:**
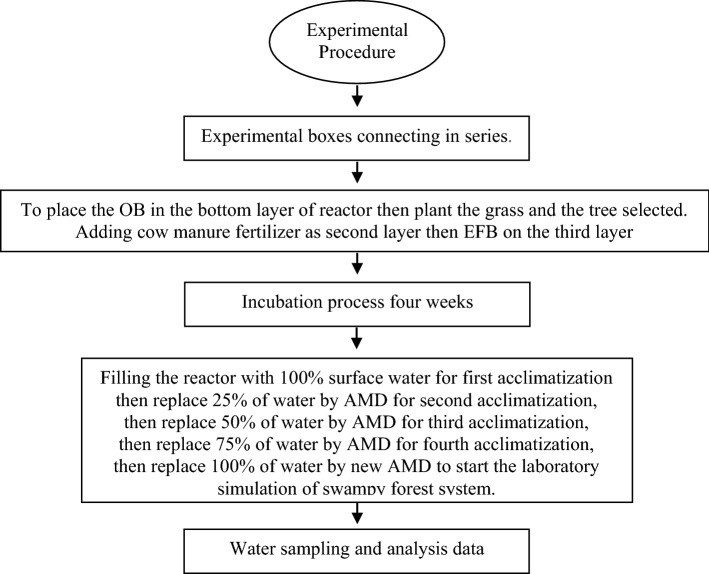
Flow chart of experimental procedure.

The treatment protocol was to place a layer of OB (250 kg) in the bottom layer in each reactor, then plant the grass (18 clumps each species) and the tree selected (three seeds each species), based on the lay-out mentioned in Fig. 1, then continue treatment by adding cow manure fertilizer (CMF) as an organic fertilizer to support grass and tree plantings, and finally adding empty fruit bunches (EFB) on the second layer^17^. Each reactor was then incubated for four weeks. The flow chart of experimental procedure as mentioned in Fig. 2.

**Figure 3:**
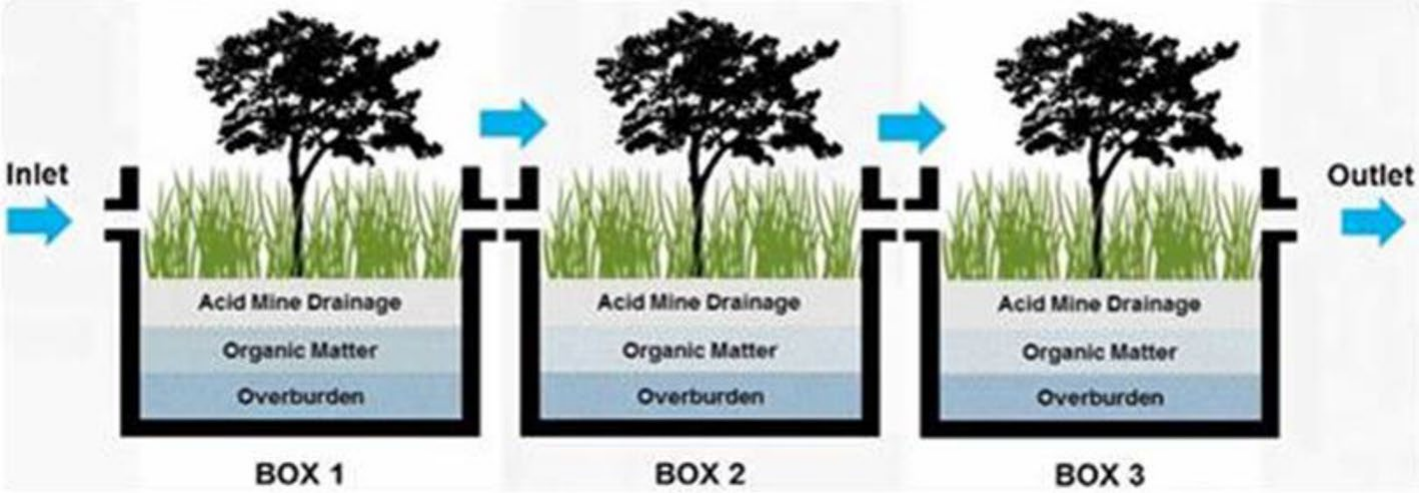
The AMD flows into the inlet, then box-1 discharges into box-2, then into box-3 and the outlet. The pH was monitored in each box from day 1 until day 5 and other threshold parameters were monitored at the inlet and the outlet on day 5, 10, 15, 30 only.

After the incubation process was completed, the experiment was continued by slowly filling the reactor with surface water (collecting rainwater until the water level of each reactor had an average height of 15 cm from the top OB layer) and allowing it to acclimate for seven days. Then, 25% of the remaining water in each reactor was drained via a tap at the bottom and replaced with AMD for the second acclimation of another seven-day process; this step was repeated for a third time by draining 50% of the remaining water and for the fourth acclimation process, by draining 75% of the remaining water. When the total acclimatization period was completed, all (100%) the water in the reactor was replaced again with full AMD to start the treatment period^17^. The laboratory simulation experiment is illustrated in Fig. 3.

After an incubation period of four weeks, the AMD continued to flow into the experimental box and entered the inlet via the surface system, flowing to box-1, then box-2, then box-3, and finally, out of the outlet (as a simulation of public water bodies). The measurement of pH water each reactor started on the day 1 (the first day of treatment) and continued until day 30. The pH measurement was carried out in the reactor box with a pH meter. Another parameters measurement was total suspended solid (TSS) and heavy metal content in the form of Fe and Mn on the day 5, 10, 15, 30 of the treatment periods. The all methods were performed in accordance with the relevant guidelines and regulations.

### Data analysis

The parameter monitoring of the laboratory simulation experiment conformed to the threshold parameter values of the environmental quality standard ​​according to regulations (South Kalimantan Governor Regulation Number 036, year 2008 concerning Wastewater Quality Standards for Mining Activities) of pH 6—9, total suspended solid (TSS) < 200 mg L^-1^, total iron content (Fe) < 7.00 mg L^-1^, total manganese content (Mn) < 4.00 mg L^-1^ and total cadmium content (Cd) < 0.05 mg L^-1^^23^. The compliance parameter values are summarized below in Table 2. The removal efficiency of each parameter was calculated to determine the potential of pH, TSS, Fe, Mn and Cd improve to meet the threshold value based on the following equation^32^:

$$\% \;{\text{Removal}}\;{\text{efficiency = }}\frac{{(C_{ini} - C_{fin} )}}{{C_{ini} }} \times 100$$

where C *ini* represents the initial concentration of metal content, while C *fin* signifies the final concentration of metal content.

**Table 2:** Threshold value compliance parameters.

Compliance		Threshold maximum value	
Parameters	Unit	National-1	Regional-2
pH	-	6–9	6–9
Total Suspended Solids (TSS)	mg L^-1^	400	200
Iron (Fe)	mg L^-1^	7.00	7.00
Manganese (Mn)	mg L^-1^	4.00	4.00
Cadmium (Cd)	mg L^-1^	-	0.05

National-1 = Decree of Environmental Ministry of Republic Indonesia, Number 113, year 2003.

Regional-2 = Regulation of Governor of South Kalimantan, Indonesia, Number 038, year 2008.

## Results and discussion

### pH water monitoring results

**Figure 4:**
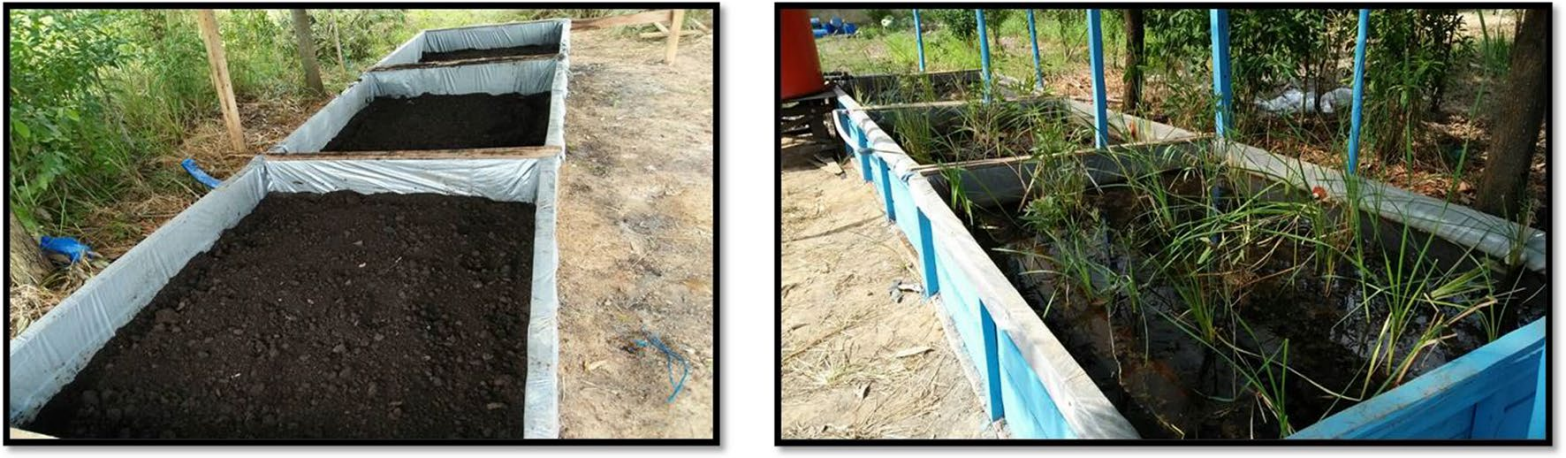
The picture of expremental box (reactor) for laboratory simulation to treat the AMD to change not comply parameter at inlet to be comply at outlet (personal documentation).

**Figure 5:**
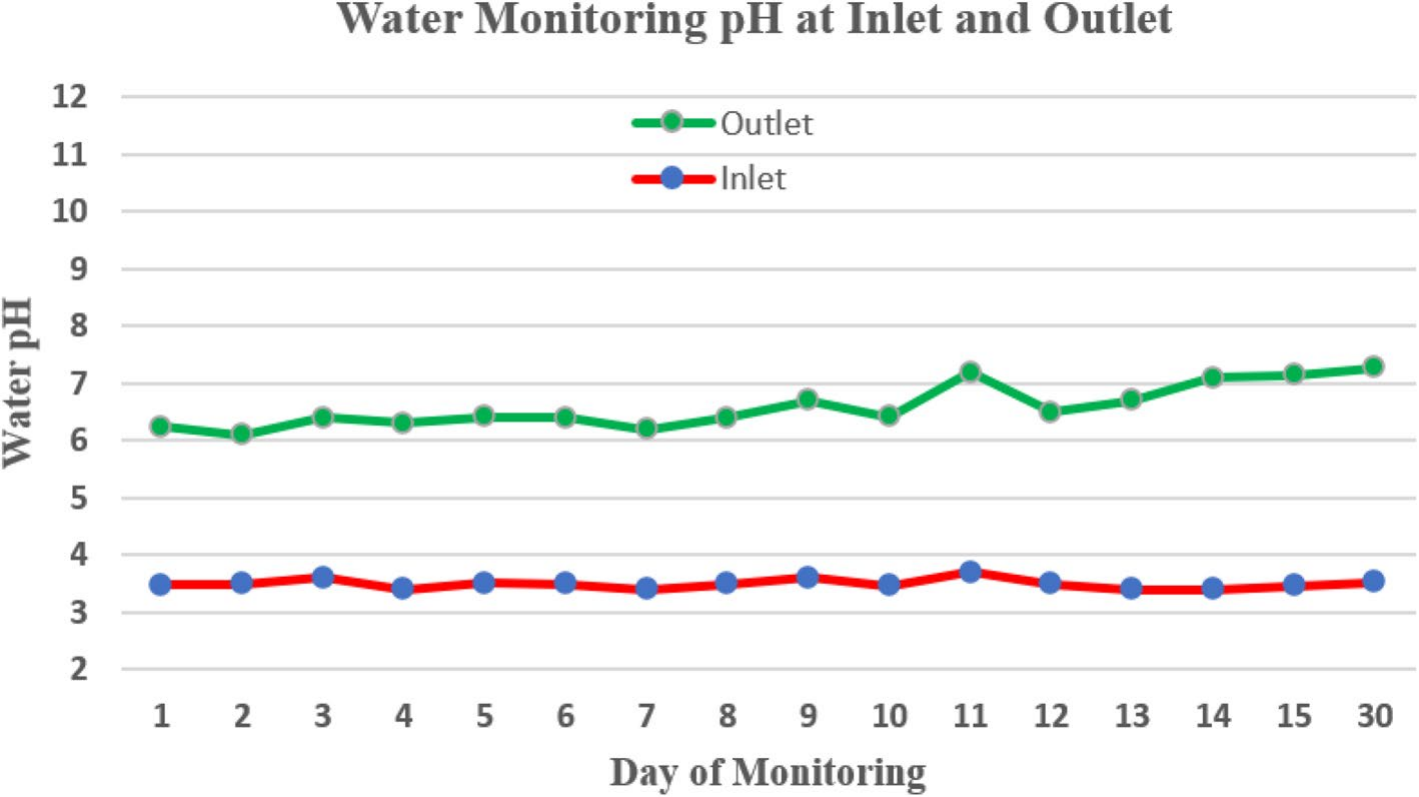
Water pH monitoring at inlet and outlet during experimental on day 1 until day 30 of treatment.

The swampy forest laboratory simulation experiment aims to obtain basic data for the development of the passive treatment for its application in the field as illustrated in Fig. 4. Observation of water pH was carried out on the fifth day of the experiments, as presented in Table 3. The AMD flowed in through the inlet and continued to overflow to box-1, then box-2, then box-3 until it flowed out (discharge) via the outlet. The average pH water observation showed that at the inlet, the pH was 3.48, which is the value that did not meet the quality standard, but the pH value continued to increase in box-1 to 4.46, box-2 to 5.75, and box-3 to 6.10 until it flowed out (discharge) via the outlet, at which point the pH was 6.24. This pH value met the quality standard value (pH 6 – 9) and was obtained with an average water flow of 1.43 m^3^ hour^-1^, a total water volume of 5.76.m^3^ and a retention time of 4.04 h. The pH monitoring data at inlet and outlet on day 1 until day 30 mentioned in Fig. 5. The pH of water increases and met with the compliance parameter.

**Table 3:** Data of pH monitoring on inlet, box-1, box-2, box-3, and outlet on day 1 until day 5 of experiment.

Experiment Number	Water pH of flowing wastewater					Total water volume	Retention time	Average water debt
	Inlet	Box-1	Box-2	Box-3	Outlet	m^3^	hour	m^3^ hour^-1^
Day-1	3.4 ^x^	3.6	5.7	5.9	6.2 ^y^	14.40	10.0	1.44
Day-2	3.5 ^x^	4.1	5.2	6.0	6.1 ^y^	3.60	2.5	1.44
Day-3	3.6 ^x^	4.6	5.8	6.3	6.4 ^y^	3.6	3.0	1.20
Day-4	3.4 ^x^	5.1	6.0	6.2	6.3 ^y^	3.6	2.5	1.44
Day-5	3.5 ^x^	4.9	6.0	6.2	6.2 ^y^	3.6	2.2	1.64
Average	3.48	4.46	5.75	6.10	6.24	5.76	4.04	1.43

Note: ^x^ = is not comply and ^y^ = is comply with regulations.

### Inlet vs. outlet comparation of compliance parameter data

The AMD that flowed into the inlet did not meet the water quality standard parameters, especially the pH and total Fe and Mn contents of the water. The average TSS and Cd data met the quality standards, as presented in Table 4. The AMD entering the inlet on day 5 showed a pH value of 3.51 or pH < 6 and after going through the swampy forest system, it increased to 6.41 or the values were in the compliance value of pH 6–9 with an increase efficiency of 82.62%. The AMD that flowed into the inlet had a low TSS value and then increased to 180 mg L^-1^ but still met the quality standard value.

**Table 4:** Data of the compliance parameter of the threshold on the inlet compared with the outlet.

Day monitoring	Parameter of compliance	Value at inlet	Value of compliance	Value at outlet	Improvement efficiency (%)
5th	pH	3.51 ^x^	6 – 9	6.41 ^y^	82.62
	TSS	15 ^y^	< 200 mg L^-1^	180 ^y^	> 200
	Fe	10.09 ^x^	< 7.0 mg L^-1^	6.43 ^y^	36.27
	Mn	12.20 ^x^	< 4.0 mg L^-1^	3.67 ^y^	69.92
	Cd	< 0.001 ^y^	< 0.05 mg L^-1^	< 0.001 ^y^	-
10th	pH	3.46 ^x^	6 – 9	6.42 ^y^	85.55
	TSS	28 ^y^	< 200 mg L^-1^	160 ^y^	> 200
	Fe	11.18 ^x^	< 7.0 mg L^-1^	6.22 ^y^	44.36
	Mn	10.85 ^x^	< 4.0 mg L^-1^	3.51 ^y^	67.65
	Cd	< 0.001 ^y^	< 0.05 mg L^-1^	< 0.001 ^y^	-
15th	pH	3.47 ^x^	6 – 9	7.14 ^y^	105.76
	TSS	35 ^y^	< 200 mg L^-1^	154 ^y^	> 200
	Fe	10.55 ^x^	< 7.0 mg L^-1^	6.33 ^y^	40.00
	Mn	11.15 ^x^	< 4.0 mg L^-1^	3.42 ^y^	69.33
	Cd	< 0.001 ^y^	< 0.05 mg L^-1^	< 0.001 ^y^	-
30th	pH	3.53 ^x^	6 – 9	7.27 ^y^	105.95
	TSS	30 ^y^	< 200 mg L^-1^	127 ^y^	> 200
	Fe	11.53 ^x^	< 7.0 mg L^-1^	5.92 ^y^	48.66
	Mn	10.55 ^x^	< 4.0 mg L^-1^	3.22 ^y^	69.48
	Cd	< 0.001 ^y^	< 0.05 mg L^-1^	< 0.001 ^y^	-

*Note: *^*x*^ = *is not comply and *^*y*^ = *is comply with regulations.*

For the metal content in AMD, the inlet showed that the total Fe and Mn did not met the quality standard values of 10.09 and 12.20 mg L^-1^, respectively, and then at the outlet, they decreased and met the quality standard value; the Fe content was 6.43 mg L^-1^ with a reduction efficiency of 36.27%, and the Mn content was 3.67 mg L^-1^ with a reduction efficiency of 69.92%. Observations of Cd do not show problems considering that it was very low below the quality standard value. Continued observing on day 10, 15, and 30, the system was able to change the not comply value to be comply.

## Discussion

### Effect of Swampy Forest System on the Changes in Threshold Parameter Value

Voids are a source of AMD with a low pH value and high metal content; this wastewater is not allowed to be released into public waters. The AMD entering the treatment of swampy forest system sees an increase in its pH value and a decrease in its metal content. The presence of organic matter in the swampy forest systems can increase the absorption, deposition, and binding of metals^29^. Most AMD has a low organic carbon content, so it requires an electron donor (hydrogen or organic compound). Oxidation reactions in the soil use inorganic carbon, while the reduction process is stimulated by organic carbon, which acts as a carbon source and electron donor^22,25^. The effect of organic matter showed different results due to different conditions during the decomposition process and the environments that support it. In anaerobic conditions, the most important role is sulfate-reducing bacteria, which thrive in environments that lack oxygen, while the decomposition of organic matter occurs under aerobic conditions that require a large amount of oxygen^10^. Flooded conditions can reduce the sulfate concentration. Sulfate-reducing bacteria take up O in the environment lacking O_2_ as a component of SO_4_^2-^ for the metabolic process of organic matter decomposition, which then produces H_2_S or becomes solid sulfate^29^. The combination of the use of organic matter, grass species and tree species are an integrated process of utilizing artificial swamp forest conditions by enriching grass and tree species as an integrated process among AMD processing and revegetation processes potential a better growth. In terms of contaminants, it can be reduced, and the area prepared for the land reclamation process by utilizing the development of bacteria in the phytoremediation process^9^. Increasing the pH of the water to a neutral condition will also help the process of plant growth^16,34^. Parameters of Eh and pH have proven very helpful in characterizing the stability of minerals in sedimentary environments that Eh–pH diagrams calculated from thermodynamic data (e.g.,^5,30^, they have not proven to be useful, in a practical sense, to individuals working with modern unlithified sediments.

Observation of the TSS parameter in this experiment showed an increase during the treatment process due to the release of OB and EFB particles, which are a group of materials that are not easily soluble in water and consist of particles that are smaller in size and heavier in mass than sediment. TSS may consist of silt, clay, metal oxides, sulfides and other inorganic particles, which can contribute to increasing turbidity, which limits light penetration for photosynthesis and visibility in water^6^.

Observations of heavy metals in the form of Fe, Mn and Cd have confirmed several previous studies that certain types of grass and tree species can absorb metal content in water. These grass species also show better performance in a constructed wetland in a previous study^35^. Types of grass such as purun grass, batibati grass, typha grass and vetiver grass can tolerate and adapt well by forming defensive colonies and are able to survive in flooded and acidic conditions^12,29^. The grass species adapted as shown by their better biomass production and efficiency to reduce the Fe and Mn^15^. which can be indicated by the grass species accumulation potential^13^. The selected tree species are also tolerant species, as their growth was not significantly disturbed. Types of galam trees, bangkal trees, longkida trees and kayuputih trees are species that are able to survive and tolerate acidic and flooded conditions^20,33^. The accumulation of certain metals in plants varies greatly among tree species, and the uptake of elements by plants mainly depends on the grass or tree species. Some types of grass and trees can limit the absorption of heavy metals and limit their movement to plant tissues through root cells, which act as the first defense at the extracellular level through root absorption and metal ion binding^34^. The combination of planting grass and tree species with local and nonlocal specifications showed the optimum results. When higher heavy metal ions accumulate in the cytosol, plants must detoxify these contaminants and minimize their toxic effects^2^ through the chelation of lignin with heavy metal ions^34^. Pitoremediation is one of the processes that helps the absorption of metals by plants and depends on the ability of roots to limit the mobility of contaminants and their availability in the soil through binding, deposition, or reduction of complexity^31^. The grass and tree species selected can absorb heavy metals through their roots and shoots by reducing the content of water in the soil through a fairly high transpiration process in the rooting area, which ultimately limits the movement of heavy metals in the soil^28^. Some root exudates can change the pH of rooting areas, which can help the deposition of heavy metals, limit their availability and reduce the level of poisoning^4^. When excess heavy metal ions accumulate in the cytosol, plants must detoxify these contaminants to minimize their toxic effects^2^.

### Scale-up the laboratory data to the field application pilot project

Laboratory simulation experiments based on combination treatments of organic matter, grass species and tree species have been determined as the basic data reference for scale-up applications in the field. The swampy forest system has demonstrated its ability to change the water quality that has not met the quality standard parameter values to water quality that is in compliance with applicable regulations^17^. The scale-up process of the basic data scale-up to the pilot project is shown in Table 5 and design for pilot project of swampy forest system as illustrated in Fig. 5.

**Table 5:** Calculation scales up process reference with the basic data of Simulation Laboratory Experiment.

Description	Laboratory simulation result data	Scale up	Pilot project design calculation on field
(Basic data)	(a)	(b)	(c = a x b)
Total volume of water	V_1_		V_2_ = (V_1_ x T x Cf_1_)
Debt of water flow in	D_1_	T (times)	D_2_ = (D_1_ x T x Cf_2_)
Retention time	R_1_		R_2_ = (R_1_ x T x Cf_3_)

Note: Cf_1_ = correction factor (CF) for water volume; Cf2 = CF for water debt; Cf3 = CF for retention time.

**Figure 6:**
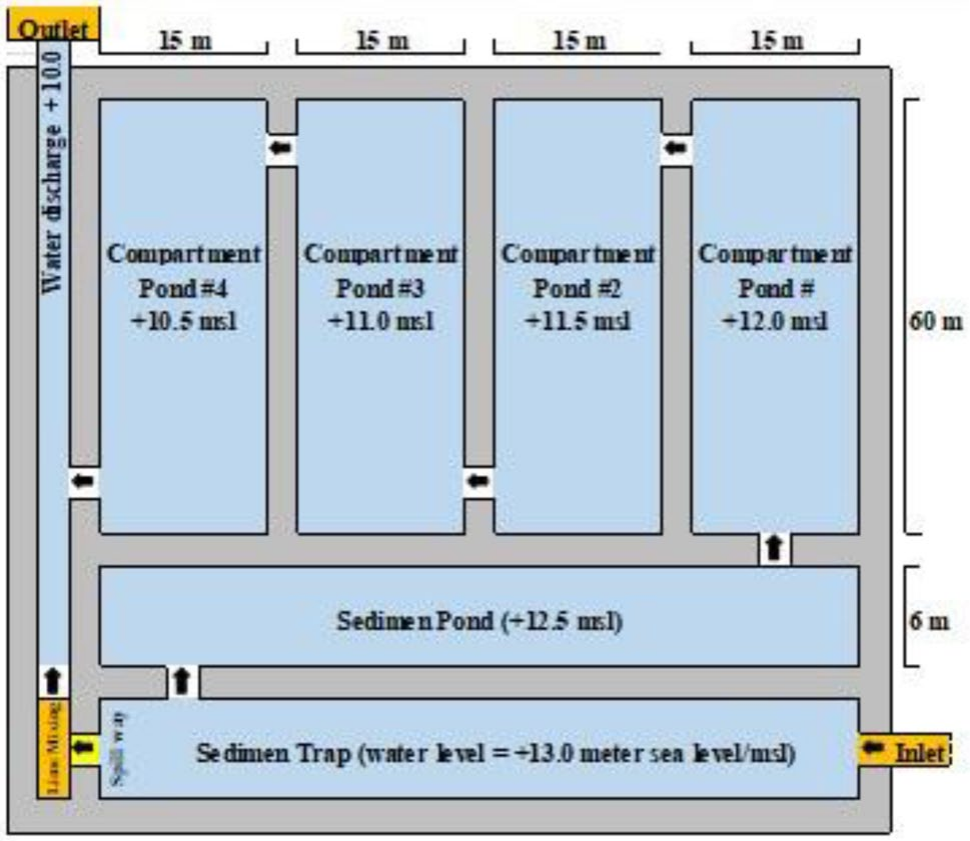
Layout design for construction swampy forest system referenced by basic data of laboratory simulation result.

The use of laboratory simulation basic data for the construction of a swampy forest system in the field. The design of construction of a swampy forest system is very dependent on the conditions of the area where the swampy forest system will be applied in the field. Considering on field conditions, the basic data obtained from laboratory simulations will be scaled up by 1.000 times which will be the location of one hectare pilot project to consider a convenient calculation. Laboratory simulation reactors that have a volume of 1.2 m^3^ of wastewater per reactor will be converted into a compartment of 900 m^3^ with a length of 60 m and a width of 15 m with water depth of 1 m. The water discharge that will flow through the swampy forest system is converted to 1,000 m^3^ day^-1^ or 41.67 m^3^ h^-1^ by a correction factor of 0.03472223 while the retention time is 108.8 h for a volume of 3600 m^3^ which is divided into four compartments. The pilot project plan in the field is designed as illustrated in Fig. 6. Conversion of basic data from laboratory simulation results converted to the field will be tested and corrected in pilot project experiments.

## Conclusion

The laboratory swampy forest system experiment showed the compliance performance for treating AMD, with an incompliance value changing to the compliance value of the threshold parameter. The basic data for scale-up reference comprises determining the capacity of the flow rate, the volume of water flowing into the swampy forest system and the retention time needed to obtain, at the end of the treatment process, a water quality that meets the quality standard parameter values before the wastewater is released to water public bodies. The swampy forest system in the data laboratory can be used as a reference when scaling up to a pilot project in which a swampy forest system is designed and constructed in the field. Compared to previous processing, the swampy forest system can reduce processing costs, increase the process of passive treatment capacity, naturally process, and more environmentally friendly because it utilizes organic waste and reduces the use of chemicals for wastewater treatment to meet the compliance threshold before release to public bodies.

## Data Availability

For data availability, all data generated or analyzed during this study are included in this manuscript.
